# Using *In Vitro* Immunomodulatory Properties of Lactic Acid Bacteria for Selection of Probiotics against *Salmonella* Infection in Broiler Chicks

**DOI:** 10.1371/journal.pone.0147630

**Published:** 2016-01-22

**Authors:** Junchang Feng, Lihong Wang, Luoxiong Zhou, Xin Yang, Xin Zhao

**Affiliations:** 1 College of Animal Science and Technology, Northwest A&F University, Yangling, Shaanxi, People’s Republic of China; 2 Department of Animal Science, McGill University, Ste. Anne de Bellevue, Quebec, Canada; East Carolina University School of Medicine, UNITED STATES

## Abstract

Poultry is known to be a major reservoir of *Salmonella*. The use of lactic acid bacteria has become one of successful strategies to control *Salmonella* in poultry. The purpose of this study was to select lactic acid bacteria strains by their *in vitro* immunomodulatory properties for potential use as probiotics against *Salmonella* infection in broiler chicks. Among 101 isolated lactic acid bacteria strains, 13 strains effectively survived under acidic (pH 2.5) and bile salt (ranging from 0.1% to 1.0%) conditions, effectively inhibited growth of 6 pathogens, and adhered to Caco-2 cells. However, their *in vitro* immunomodulatory activities differed significantly. Finally, three strains with higher *in vitro* immunomodulatory properties (*Lactobacillus plantarum* PZ01, *Lactobacillus salivarius* JM32 and *Pediococcus acidilactici* JH231) and three strains with lower *in vitro* immunomodulatory activities (*Enterococcus faecium* JS11, *Lactobacillus salivarius* JK22 and *Lactobacillus salivarius* JM2A1) were compared for their inhibitory effects on *Salmonella* adhesion and invasion to Caco-2 cells *in vitro* and their antimicrobial effects *in vivo*. The former three strains inhibited *Salmonella* adhesion and invasion to Caco-2 cells *in vitro*, reduced the number of *Salmonella* in intestinal content, spleen and liver, reduced the levels of lipopolysaccharide-induced TNF-α factor (LITAF), IL-1β, IL-6 and IL-12 in serum and increased the level of IL-10 in serum during a challenge study *in vivo* more efficiently than the latter three strains. These results suggest that *in vitro* immunomodulatory activities could be used as additional parameters to select more effective probiotics as feed supplements for poultry.

## Introduction

*Salmonella* is one of the major causes of food-borne illnesses in humans. Poultry is known to be a major reservoir of *Salmonella*. Vaccination and bio-security have been adopted to control *Salmonella* in poultry production. In addition, lactic acid bacteria (LAB) based probiotics have been used to control *Salmonella* in poultry [1]. LAB probiotics mainly consist of *Lactobacillus*, *Pediococcus* and *Enterococcus*. These bacteria can competitively exclude pathogens by adherence to the host intestinal epithelium [2], enhance immune functions and improve the intestinal barrier of hosts [3,4].

Probiotics have usually been selected using conventional selection parameters including tolerance to acids and bile salt, antimicrobial activities, adhesion to epithelial cells and inhibition of pathogen adhesion to epithelial cells [5]. Some probiotics screened by conventional indicators have been shown to be beneficial to poultry health. For example, a single dose of *Lactobacillus salivarius* reduced the rate of *Salmonella* infection in 3-day old chicks [6]. Oral administration of 10^6^ or 10^8^ cfu of *Lactobacillus*-based probiotic culture significantly reduced *Salmonella* Enteritidis recovered from cecal tonsil of neonatal chicks [7]. However, other probiotics selected by conventional indicators are not effective. For example, Yamawaki et al. [8] showed that *L*. *acidophilus*, *L*. *fermentum* and *L*. *salivarius* did not decrease *Salmonella* Enteritidis colonization of chick ceca. Also, pre-treatment with *Lactobacillus acidophilus*, *Bifidobacterium bifidum* and *Streptococcus faecalis* did not change *IL-6* and *IL-10* gene expression in cecal tonsils of chicks [9]. The inconsistent results demonstrate the necessity of additional parameters to screen LAB for probiotics.

*In vitro* immune-modulating effects of LAB have received increasing attention as potential selection parameters for probiotics recently. The ability of LAB to induce IL-12 production was used to examine the immune-enhancing activity of LAB [10]. In addition, Tsai et al. [2] evaluated the ability of 12 LAB strains to stimulate production of TNF-α by mouse RAW 264.7 macrophage cells before they used LAB in a *Salmonella* challenge study in mice. Similarly, Chen et al. [4] evaluated production of TNF-α by the same mouse RAW 264.7 macrophage cells for selection of LAB to be used in a *Salmonella* challenge study in day-old chicks. Chen et al. [3] further included production of IL-12 protein by mouse RAW 264.7 macrophage cells as a parameter to select LAB for a *Salmonella* challenge study in mice. Gene expression of several cytokines by real-time quantitative PCR was significantly increased when mononuclear cells isolated from spleens of chicks were co-cultured with probiotic LAB for poultry [11]. However, measurement of cytokine production in chicks has been hampered by lack of commercial antibodies. Considering the species difference, we hypothesized that it would be better to use tissues or cells from chickens, instead of mouse RAW 264.7 macrophage cells, to select LAB as probiotics in chicks. TNF-α has not been found in the chicken genome. Instead, lipopolysaccharide-induced TNF-α factor (LITAF) has been cloned (Hong et al., 2006) and is one of the most important monitoring indexes for evaluating inflammatory response. With preparation of specific antibodies against LITAF and IL-12 in chicks in our laboratory, therefore, the objective of this study was to test the hypothesis whether expression levels of LITAF and IL-12 at the protein levels in spleen mononuclear cells *in vitro* could be used as additional selection parameters to select LAB as candidate probiotics for chickens.

## Materials and Methods

### Ethics statement

The study was approved by Institutional Animal Care and Use Committee of Northwest A&F University (Permit Number: NWAFAC1019). All chicks were euthanized via cervical dislocation, and all efforts were made to minimize suffering.

### Bacterial isolates, culture media, and growth conditions

Lactic acid bacteria were isolated from intestinal contents of healthy free-range laying hens, according to the method described by Brisbin et al. [12]. Briefly, laying hens were purchased from Yangling, Shaanxi, China, and euthanized via cervical dislocation. Two hundred fifty mg of intestinal content from duodenum, jejunum, ileum or cecum were diluted with 10 ml phosphate-buffered saline (PBS, pH 7.4) and plated onto DeMan, Rogosa, and Sharpe (MRS) plates (Becton Dickinson, Mississauga, ON, Canada) for selecting lactic acid bacteria, [13]. The plates were grown at 37°C under anaerobic conditions (85% N_2_, 10% CO_2_, and 5% H_2_) for 48 h, and individual colonies were selected and inoculated into MRS broth, cultured at 37°C under anaerobic conditions for 18 h and subcultured twice. The V3 region of the 16S rRNA gene was amplified by PCR using the specific primers and sequenced as described in a previous study [13] and sequence comparisons were performed using the Basic Local Alignment Search Tool (BLAST) program (http://www.ncbi.nlm.nih.gov/BLAST/).

Pathogenic bacteria used in this study to measure antibacterial activities of probiotics included *Staphylococcus aureus* ATCC 29213, *Escherichia coli* K88, 25922 and 1569, *Salmonella* Enteritidis ATCC 13076 and *Salmonella* Typhimurium ATCC 14082. To culture these bacterial strains, one colony of each strain was inoculated into 5 ml tryptic soy broth and incubated with shaking at 37°C for 12 h.

### Culture of cell lines

Caco-2 cells (ATCC HTB-37), a human colon adenocarcinoma cell line, were maintained in Dulbecco’s modified Eagle’s medium (DMEM)/F12 media (Gibco, USA) supplemented with 10% (v/v) inactivated (30 min, 56°C) fetal bovine serum, 20 U/ml penicillin and 100 μg/ml streptomycin. Cells were cultured at 37°C in a 5% CO_2_/95% air atmosphere using a humidified CO_2_ incubator. Cells were used at post-confluence after 21 days of culture. For adhesion assays as well as inhibition of intestinal cell adhesion and invasion by pathogens, monolayers of Caco-2 cells were prepared in 24-well tissue culture plates (Costar 3524, Corning Inc. NY, USA). Cells were seeded at a concentration of 5 × 10^4^ cells/ml in the DMEM-F12 medium without penicillin and streptomycin.

### Preparation of spleen mononuclear cells

Spleens were harvested sterile from four adult Arbor Acres chickens, which were obtained from Yangling, Shaanxi, China. After euthanizing via cervical dislocation, the spleens were rinsed in 1 × Hanks’ balanced salt solution (HBSS) and then minced with sterile scalpels. The tissue was further disrupted with the flat end of a 10-ml syringe plunger and filtered through a 40-μm nylon cell strainer to obtain a single-cell suspension. The suspension was then overlaid onto a Histopaque-1077 (Sigma, Oakville, ON, Canada) density gradient and centrifuged at 400 × ***g*** for 30 min. Mononuclear cells at the interface were collected and washed twice in 1 × HBSS and then suspended in RPMI 1640 (RPMI containing 10% fetal bovine serum, 2% chick serum, 0.146 g L-glutamine, 1.6 mM 2-mercaptoethanol). Cells were counted by the trypan blue dye exclusion assay before being resuspended in RPMI 1640 [12].

### Resistance of LAB to acid and bile and antimicrobial activities of LAB

To evaluate acid resistance among the LAB strains, methods described by Tsai et al. [2] were used. Effects of bile salts on the growth of LAB cells were studied by a method modified from that of Osmanagaoglu et al. [14]. Briefly, for determining the acid tolerance, 150 μl of each culture containing about 10^8^−10^9^ cfu/ml of LAB suspension was added to 4.85 ml MRS that had been adjusted to pH 2.5 by 0.1 N HCl. To test the bile salt resistance, the same inoculum was added to normal MRS supplemented with 0.1%, 0.3%, 0.5% or 1.0% bile salts (Sigma, Saint Louis, Missouri, USA). Each mixture was incubated at 37°C for 3 h. After incubation, viable bacterial counts were determined by plating serial dilutions (with PBS, pH 7.4) on the MRS agar under anaerobic conditions at 37°C for 48 h. These assays were performed in triplicate for each of 3 independent experiments.

Antibacterial activities of LAB strains were studied using an agar diffusion test. Strains of LAB were grown overnight (20 h) in MRS broth at 37°C. Pathogenic bacterial strains were grown in the Luria–Bertani (LB) agar. Then, wells were hollowed out of the LB plates using an Oxford cup and 200 μl of the spent culture suspension (LAB-SCS) was added into each well. The culture was incubated at 37°C for 14 h before determination of antimicrobial activities. LAB strains with inhibition zones <11, 11–16, 17–22 and >23 mm were classified as strains of no (-), mild (+), strong (++), or very strong (+++) inhibition, respectively [2].

### Adhesion of LAB to intestinal cell lines

The adhesion study of LAB strains was performed by following the procedures described by Bianchi et al. [15] and Tsai et al. [2]. LAB strains were stained with fluorescein isothiocyanate (FITC, Sigma, USA) and kept in darkness at 37°C for 2 h, then washed three times with antibiotic-free PBS solution (pH 7.4) to remove the unlabeled FITC and resuspended with PBS. One hundred μl of each suspension (5×10^8^ cfu/ml) was transferred to the 24-well multidish containing the Caco-2 cells and incubated for 2 h. After incubation, non-adherent bacteria were removed by washing three times with PBS. Two hundred μl of trypsin/EDTA-Na_2_ were used to digest the cells and adherent bacteria for 5 min before 600 μl of PBS were added into the wells. After mixing, a 200 μl mixture containing cells and bacteria was added to 96 well plates. This fraction contained lysed bacteria attached to Caco-2 cells or within Caco-2 cells and it was reported as the adherent fraction. The fluorescence was read on a Perkin-Elmer LS55 Spectrophotometer (λ_ex_ = 492 nm; λ_em_ = 517 nm). Six independent experiments were performed for each microbial strain on Caco-2 cells. The results of adhesion assay were expressed as the adhesion percentage of adherent bacteria over added bacteria per well.

### Production of polyclonal antibodies against LITAF, IL-1β, IL-6, IL-10 and IL-12

Polyclonal antibodies against LITAF, IL-1β, IL-6, IL-10 and IL-12 (antigens) were obtained by immunizing female New Zealand white rabbits (2–2.5 kg body weight) with LITAF, IL-1β, IL-6, IL-10 and IL-12 as previously described elsewhere [16].

In order to prepare antigens for immunization, plasmids pET32a-LITAF, pET32a-IL-1β, pET32a-IL-6, pET32a-IL-10 and pET21a-IL-12 were prepared. Polymerase chain reactions (PCR) were performed on total cDNA of chick using specific primers:

LITAF-F 5’-GGGGTACCATGTCTGCTCCTAGTGGCTTT-3’ sense primer and LITAF-R 5’-CCAAGCTTCTACGCTCCTGACTCATAGCAGAG-3’ antisense primer (GenBank: AY765397); IL-1β-F 5’-GGGGTACCATGGCGTTCGTTCCCG-3’ sense primer and IL-1β-R 5’-CCAAGCTTTCAGCGCCCACTTAGCTT-3’ antisense primer (GenBank: DQ393267); IL-6-F 5’-GGGGTACCGGAGAGGTTGGGCTGGAG-3’ sense primer and IL-6-R 5’-CCAAGCTTTCAGGCACTGAAACTCCTGG-3’ antisense primer (GenBank: HM179640); IL-10-F 5’-GGGGTACCTGCTTGGAGCCCACCT-3’ sense primer and IL-10-R 5’-CCAAGCTTTCACTTCCTCCTCCTCATCA-3’ antisense primer (GenBank: EF554720). IL-12-F 5’-GGGGATCCAAAGAGCCAAGCAAGACG-3’ sense primer and IL-12-R 5’-CCAAGCTTGAAAGTCAAAGGGAAGTAGGA-3’ antisense primer (GenBank: DQ202328). The recombinant plasmids were subsequently introduced into BL21 (DE3) or Transetta (DE3). After induction by IPTG, the target proteins were purified. The rabbits were initially immunized with two intramuscular injections of LITAF, IL-1β, IL-6, IL-10 or IL-12 (300 μg antigens) dissolved in 1 ml ice-cold (4°C) PBS and complete Freund’s adjuvant in a ratio of 1:1. Immunization was repeated three times at 2 week intervals using the same amounts of antigens in the incomplete Freund’s adjuvant.

### Assays for LITAF and IL-12 production by spleen mononuclear cells after stimulation with LAB strains

Spleen cells were cultured in triplicate, at a density of 1×10^6^ cells/ml of RPMI-1640 medium without penicillin or streptomycin, in 24-well tissue culture plates. LAB cells were centrifuged at 8,000 ***g*** for 5 min and the pellet was resuspended in RPMI-1640 medium containing spleen cells to a final concentration from 10^8^ to 10^9^ cfu/ml. Lipopolysaccharide (10 μg/ml) from *E*. *coli* O26:B6 (Sigma, USA) was used as a positive control. After 24 h and 48 h, LITAF and IL-12 produced in these culture supernatants were analyzed.

Cytokines were measured using an enzyme-linked immunosorbent assay (ELISA) method. Ninety-six-well Immuno-Maxisorp plates (Nunc) were coated with polyclonal antibodies for LITAF and IL-12 (1: 1,000) in coating buffer (0.05 M Carbonate Buffer, pH 9.6) overnight at 4°C. Plates were blocked and washed. Culture medium was added to the plates and they were incubated for 2 h at room temperature. Plates were then washed again, and goat-anti-rabbit secondary antibody conjugated horseradish peroxidase (1: 20,000) was added, followed by incubation for 1 h at room temperature and another wash. The chromogenic reactions were developed with the 3, 3’, 5, 5’-tetramethylbenzidine substrate at 37°C for 30 min. The reactions were terminated with 50 μl of 2N H_2_SO_4_ and the absorbance at A_450 nm_ was measured. Equivalent levels of LITAF and IL-12 were calculated by comparison with reference curves generated using LITAF and IL-12 standards. The results were expressed as the concentration of the cytokines in the culture medium (ng/ml).

### Inhibition of *Salmonella* adhesion and invasion of Caco-2 cells by LAB strains

Each LAB strain (5×10^7^ cfu per well) was added to Caco-2 cells in a fresh tissue culture medium without Penicillin-Streptomycin and incubated at 37°C for 2 h in a 5% CO_2_/95% air atmosphere incubator before 2 h of incubation with 100 μl of FITC labeled *Salmonella* (5×10^8^ cfu/ml). *Salmonella* were labeled with Fluorescein isothiocyanate (FITC, Sigma, USA) in darkness at 37°C for 2 h. After incubation with FITC labeled *Salmonella*, non-adherent bacteria were washed away three times with PBS.

To determine the relative adhesion of *Salmonella* to Caco-2 cells, one 24-well multidish was added with 200 μl trypsin to digest the cells and adherent bacteria for 5 min before adding 600 μl of PBS. After mixing, 200 μl of mixture containing cells and bacteria were added to each well of 96-well plates to measure the strength of fluorescence. To determine the relative invasion of *Salmonella* to Caco-2 cells, another 24-well multidish was lysed with 200 μl of 1% Triton X-100 for 10 min before trypsin digestion. Six independent experiments were performed for each LAB strain.

The relative adhesion or invasion of *S*. Enteritidis ATCC13076 to Caco-2 cells was expressed as a percentage using the following formula: relative of adhesion or invasion = 100 x A1/A2, where A1 and A2 were the percentages of adhesion or invasion by *S*. Enteritidis ATCC13076 in the presence and absence of LAB strains, respectively.

### Challenge study

The challenge study was performed by following the procedures described by Chen et al. [4] with few modifications. One hundred ninety-two hatched 1-d-old healthy Arbor Acres male broilers were obtained from a commercial hatchery at Xianyang, Shaanxi, China. Chicks were reared in two layer metal cages, with an average stocking density of 16.7 birds per square meter, and the brooding temperature was 31 to 33°C throughout the experiment. Chicks had free access to water and a commercial starter diet without supplementation of antibiotics. The chicks were randomly divided into 8 groups (6 repeats/group, 4 chicks/repeat): In the negative control group (group 1), the chicks were only given sterile PBS buffer (pH 7.4) (0.2 ml/chick) via the intragastric route once every day throughout 4 experimental days. In the positive control group (group 2), chicks were challenged with *Salmonella* on d4 (0.2 ml/chick, 10^8^ cfu per 0.2 ml) and were given sterile PBS buffer (pH 7.4) (0.2 ml/chick) during d1-d3. For treatment groups 3–5, 3 LAB strains with higher *in vitro* immunomodulatory properties (*L*. *plantarum* PZ01, *L*. *salivarius* JM32 and *P*. *acidilactici* JH231) were used, while 3 LAB strains with lower *in vitro* immunomodulatory properties (*E*. *faecium* JS11, *L*. *salivarius* JK22 and *L*. *salivarius* JM2A1) were used for treatment groups 6–8. For treatment groups 3–8, chicks were gavaged with LAB (0.2 ml/chick, 10^9^ cfu per 0.2 ml) once every day for 3 consecutive days, then challenged with *Salmonella* Enteritidis ATCC 13076 on d4 (0.2 ml/chick, 10^8^ cfu per 0.2 ml).

Broiler chickens were euthanized via cervical dislocation. Samples for blood, spleens, livers and cecum contents of six randomly selected chicks in each group were taken at 1, 3 and 5 days post *Salmonella* challenge. *Salmonella* Enteritidis ATCC 13076 was selected for the challenge study, due to the invasive characteristic previously described by Dawoud et al. [17].

### Assessment of LITAF, IL-1β, IL-6, IL-10 and IL-12 in chick serum and enumeration of the *Salmonella* cells invaded in chick liver and spleen, and colonized in the cecum

For all groups, blood samples were collected from the carotid artery of 6 chicks at 1, 3 and 5 days post *Salmonella* challenge. Blood serum was obtained after incubation for 1 h at room temperature followed by 2,000 ***g*** for 10 min. Sera were stored at -80°C until tested. The cytokines LITAF, IL-1β, IL-6, IL-10 and IL-12 were measured by the ELISA method with preparation of specific antibodies of LITAF, IL-1β, IL-6, IL-10 and IL-12 in our laboratory. Samples for spleens, livers and cecum contents of six randomly selected chicks in each group were taken at 1, 3 and 5 days post *Salmonella* challenge. *Salmonella* that had invaded the spleens and livers and colonized in the cecum of chicks were enumerated by cfu method [4]. Spleens and livers were homogenized and without serial dilution, and cecum contents were diluted with PBS (pH 7.4). All the samples were incubated on selected Brilliant Green agar (Difco) containing 50 μg/ml novobiocin (Sigma, USA) for 24 h at 37°C before counting the number of *Salmonella*.

### Statistical analysis

All results were expressed as mean ± SD from at least three independent experiments. Statistical analysis was performed using the SPSS for Windows version 17.0 (Chicago, IL, USA). Data were subjected to one-way ANOVA and, where appropriate, the Scheffe test was used for comparison of means. Differences were considered to be statistically significant when the *P* value was <0.05.

## Results

### Resistance of LAB to acid and bile, antimicrobial activity and *in vitro* adhesion assay to Caco-2 cells

Among the 101 isolated LAB strains, 13 strains were better than the remaining 88 LAB strains to survive following exposure to acid (pH 2.5) or bile salts for 3 h (Table 1). These 13 bacteria had a survival rate of over 77% after 3 h at pH 2.5. With the presence of 1.0% of bile salts, the survival rates were more than 60.0% for these 13 strains. Bile salts at the 0.1% level had no significant effect on bacterial survival. As shown in Table 2, the above-mentioned 13 LAB strains were able to inhibit the growth of the pathogenic bacteria. All 13 LAB strains had strong inhibition against *Escherichia coli* except JM31, JK22, JK231 and JS11 which exhibited mild inhibition against ATCC K88. Similarly, all 13 LAB strains had strong inhibition against *Salmonella* and *Staphylococcus aureus* except JM 241 and JH231 which exhibited mild inhibition against *Staphylococcus aureus* and *Salmonella* Typhimurium.

**Table 1:** Resistance to acid and bile of the 13 LAB strains.

Strains^a^	Resistance to acid (% growth)	Resistance to bile (% growth)			
	pH 2.5	0.1%	0.3%	0.5%	1.0%
*P*. *pentosaceus* JS233	78.9 ± 4.5 *	98.5 ± 3.5	79.2 ± 3.7 *	77.6 ± 4.6 *	63.0 ± 5.8 *
*L*. *salivarius* JM41	80.4 ± 5.0 *	100 ± 3.5	82.1 ± 3.8 *	78.4 ± 5.1 *	64.7 ± 5.2 *
*L*. *plantarum* PZ01	79.6 ± 1.9 *	97.0 ± 3.3	80.5 ± 3.2 *	77.5 ± 4.3 *	64.0 ± 4.3 *
*L*. *salivarius* JK21V	79.1 ± 4.8 *	100 ± 3.2	80.2 ± 3.2 *	78.2 ± 4.1 *	62.9 ± 3.9 *
*P*. *acidilactici* JM241	78.6 ± 1.6 *	94.2 ± 3.7	80.8 ± 3.7 *	74.9 ± 4.9 *	62.7 ± 5.3 *
*L*. *salivarius* JM31	78.9 ± 3.2 *	93.1 ± 3.4	80.4 ± 3.5 *	73.5 ± 7.2 *	64.3 ± 7.7 *
*L*. *salivarius* JS2A	81.7 ± 5.2 *	99.4 ± 3.2	81.8 ± 3.8 *	72.4 ± 6.5 *	60.0 ± 6.8 *
*L*. *salivarius* JM14	77.9 ± 1.4 *	97.9 ± 3.5	81.3 ± 3.5 *	70.3 ± 3.3 *	62.7 ± 4.9 *
*L*. *salivarius* JK22	81.4 ± 1.8 *	98.8 ± 3.2	80.7 ± 3.1 *	70.0 ± 4.2 *	61.4 ± 5.6 *
*L*. *salivarius* JM2A1	77.7 ± 2.8 *	98.2 ± 2.6	80.5 ± 3.2 *	78.7 ± 3.5 *	62.2 ± 3.1 *
*L*. *salivarius* JM32	80.2 ± 1.6 *	99.2 ± 4.1	78.8 ± 4.1 *	77.9 ± 5.1 *	64.0 ± 4.8 *
*P*. *acidilactici* JH231	81.2 ± 1.8 *	93.7 ± 2.8	80.0 ± 3.1 *	73.7 ± 6.2 *	63.4 ± 5.2 *
*E*. *faecium* JS11	82.3 ± 2.8 *	93.4 ± 3.5	81.4 ± 4.5 *	74.8 ± 3.0 *	65.3 ± 6.5 *

^a^ Values are means ± standard deviation from three experiments. The control was 100%.

* Considered significantly different from the control (*P*<0.05).

**Table 2:** Antimicrobial activities of lactic acid bacteria against the growth of pathogenic bacteria *in vitro*.

LAB strains	*Escherichia coli* (EC)			*S*. *aureus* ATCC 29213	*S*. Enteritidis ATCC 13076	*S*. Typhimurium ATCC 14082
	ATCC K88	ATCC 1569	ATCC 25922			
*P*. *pentosaceus* JS233	++*	++	++	++	++	++
*L*. *salivarius* JM41	++	++	++	++	++	++
*L*. *plantarum* PZ01	++	++	++	++	++	++
*L*. *salivarius* JK21V	++	++	++	++	++	++
*P*. *acidilactici* JM241	++	++	++	+	++	+
*L*. *salivarius* JM31	+	++	++	++	++	++
*L*. *salivarius* JS2A	++	++	++	++	++	++
*L*. *salivarius* JM14	++	++	++	++	++	++
*L*. *salivarius* JK22	+	++	++	++	++	++
*L*. *salivarius* JM2A1	++	++	++	++	++	++
*L*. *salivarius* JM32	++	++	++	++	++	++
*P*. *acidilactici* JH231	+	++	++	+	++	+
*E*. *faecium* JS11	+	++	++	++	++	++

* LAB strains with inhibition zones 11–16 mm and 17–22 mm were classified as strains of mild + and strong ++ inhibition, respectively.

All 13 tested strains were able to adhere to Caco-2 cells with different adhesion activities (Table 3). JS233, JM41, PZ01 and JK21V showed the strongest adherence to Caco-2 cells (from 19.67 to 10.94%), while JS11 was the least effective one to adhere to Caco-2 cells (2.24 ± 0.24%).

**Table 3:** Adhesion of lactic acid bacteria to Caco-2 cells.

Strains	Adhesion (%)* (mean ± S.D)
*P*. *pentosaceus* JS233	19.67 ± 0.48^a^
*L*. *salivarius* JM41	15.47 ± 0.22^b^
*L*. *plantarum* PZ01	11.23 ± 0.70^c^
*L*. *salivarius* JK21V	10.94 ± 0.79^c^
*P*. *acidilactici* JM241	6.49 ± 0.58^d^
*L*. *salivarius* JM31	6.35 ± 0.24^d^
*L*. *salivarius* JS2A	5.37 ± 0.37^e^
*L*. *salivarius* JM14	5.30 ± 0.63^e^
*L*. *salivarius* JK22	5.26 ± 0.64^e^
*L*. *salivarius* JM2A1	5.24 ± 0.46^e^
*L*. *salivarius* JM32	4.82 ± 0.19^e^
*P*. *acidilactici* JH231	4.45 ± 0.27^e^
*E*. *faecium* JS11	2.24 ± 0.24^f^

* Fluorescence values were the ratio between adherent bacteria and added bacteria.

^a-f^ Different superscripts indicate significant differences among different strains (*P*<0.05).

### LITAF and IL-12 production by spleen mononuclear cells in response to recall antigen stimulation

**Fig 1:**
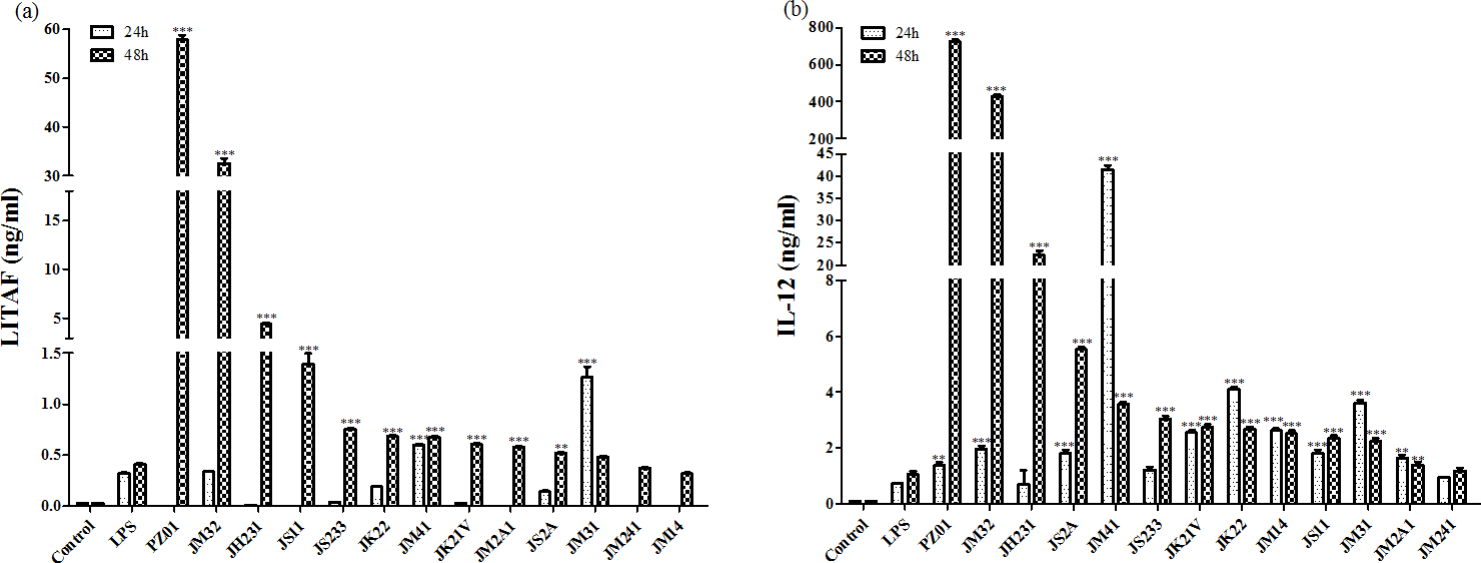
(A) LITAF, (B) IL-12 production by spleen mononuclear cells after incubation with different LAB strains. Spleen cells (1×10^6^ cells/ml) were cultured in a RPMI-1640 medium without penicillin or streptomycin. The RPMI-1640 medium alone was used as a negative control, while LPS (lipopolysaccharide, 10 μg/ml) was used as a positive control. LAB (10^8^ to 10^9^ cfu/ml) were cultured with spleen cells. After 24 h and 48 h of culture, LITAF and IL-12 produced in the culture supernatants were analyzed. Each value represents the mean value ± SD from three independent experiments. * indicates significant differences in comparison with the positive control (*: *P*<0.05).

Except JM31, JM241 and JM14, the remaining strains enhanced LITAF production more than the positive control at 48 h post-treatment (*P*<0.05), especially PZ01 (57.81 ng/ml), JM32 (32.64 ng/ml) and JH231 (4.44 ng/ml). Only JM31 and JM41 produced more LITAF than the positive control group after co-incubation with spleen mononuclear cells for 24 h (*P*<0.05) (Fig 1A). At 24 h post-treatment, *Pediococcus* spp. (JH231, JS233 and JM241) did not increase IL-12 production, while the other LAB strains significantly induced IL-12 production in comparison with the LPS positive control group (*P*<0.05). All 13 strains except JM241 enhanced IL-12 production more than LPS after incubation for 48 h (*P*<0.05). The highest producers of IL-12 were PZ01 (724.85 ng/ml), JM32 (427.63 ng/ml) and JH231 (22.25 ng/ml) (Fig 1B). These same 3 strains were also the highest producers of LITAF. Thus, they were retained for further assays. Three other strains (JS11, JK22 and JM2A1) were randomly selected to represent lower producers of LITAF and IL-12 for subsequent assays. The remaining 7 LAB strains were not studied further.

### Inhibition of pathogen adhesion and invasion to Caco-2 cells by LAB

**Fig 2:**
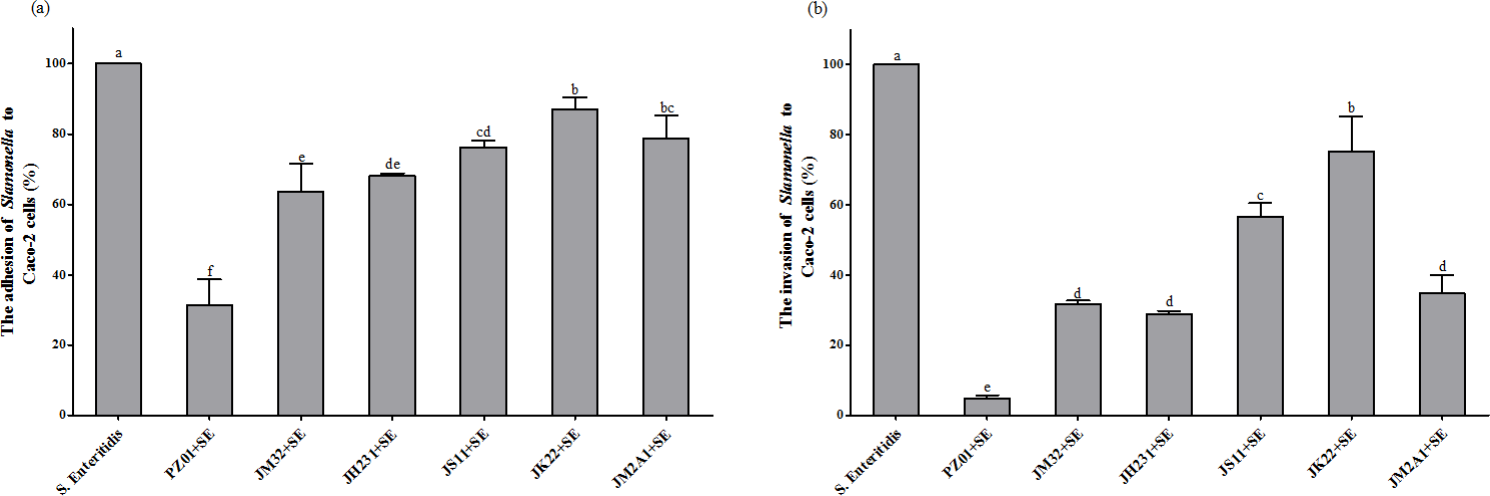
Effects of LAB culture on the adhesion and invasion of Caco-2 cells by *Salmonella* Enteritidis. The relative adhesion or invasion of *S*. Enteritidis ATCC13076 to Caco-2 cells was expressed as a percentage using the following formula: relative adhesion or invasion = 100 x A1/A2, where A1 and A2 were the percentages of adhesion or invasion by *S*. Enteritidis ATCC13076 in the presence and absence of LAB strains, respectively. Each value represents the mean value ± SD from six trials. Different letters above bars indicate significant differences among treatments within each sampling day (*P*<0.05).

The competitive inhibition of adhesion and invasion of *Salmonella* Enteritidis to Caco-2 cells by 6 probiotic strains was shown in Fig 2A and 2B, respectively. Strain PZ01 displayed the strongest inhibition of *Salmonella* Enteritidis adhesion and invasion to Caco-2 cells (Fig 2). On average, 3 strains with higher *in vitro* immunomodulatory properties reduced adhesion by 45.6% and invasion by 78.3%, while 3 strains with lower *in vitro* immunomodulatory properties reduced adhesion only by 19.4% and invasion only by 44.4%.

### Inhibitory effects of selected LAB strains on the invasion and colonization of *Salmonella in vivo*

In order to determine whether LAB strains with higher *in vitro* immunomodulatory properties had better inhibitory effects against *Salmonella in vivo*, a *Salmonella* challenge study was performed. As shown in Table 4, all 6 strains significantly decreased *Salmonella* Enteritidis in cecum content on sampling time points, compared with the positive control (*P*<0.05). On average, 3 strains with higher *in vitro* immunomodulatory properties reduced the bacterial counts by 88.37%, 94.93% and 96.63%, while 3 strains with lower *in vitro* immunomodulatory properties reduced the bacterial counts only by 61.52%, 73.12% and 76.24% on d1, d3 and d5 post-infection, respectively.

**Table 4:** Effects of oral administration of 6 different lactic acid bacteria on reduction of *Salmonella* cells recovered from the livers, spleens, and cecal content of chicks.

Item	Spleen	Liver	Cecum content
	cfu /organ	cfu /0.3 g	cfu (×10^4^)/0.25 g
**1 d Post-infection**			
*Salmonella* only	100.67 ± 5.72^a^	91.67 ± 8.16	237.28 ± 14.07^a^
PZ01 + *Salmonella*	ND	ND	26.00 ± 4.75^f^
JM32 + *Salmonella*	ND	ND	19.69 ± 1.53^f^
JH231 + *Salmonella*	ND	ND	37.13 ± 3.89^e^
JS11 + *Salmonella*	ND	ND	132.41 ± 10.82^b^
JK22 + *Salmonella*	58.33 ± 4.08^b^	ND	59.42 ± 7.17^d^
JM2A1 + *Salmonella*	ND	ND	82.03 ± 9.21^c^
**3 d Post-infection**			
*Salmonella* only	148.33 ± 17.80^a^	207.67 ± 9.63^a^	245.04 ± 13.49^a^
PZ01 + *Salmonella*	ND	ND	12.63 ± 1.70^c^^d^
JM32 + *Salmonella*	ND	ND	6.52 ± 1.16^d^
JH231 + *Salmonella*	ND	ND	18.13 ± 3.34^c^
JS11 + *Salmonella*	66.33 ± 8.16^b^	58.67 ± 8.98^c^	67.50 ± 5.00^b^
JK22 + *Salmonella*	126.67 ± 10.80^a^	158.33 ± 14.72^b^	58.98 ± 8.49^b^
JM2A1 + *Salmonella*	131.37 ± 16.33^a^	171.67 ± 8.16^b^	71.13 ± 10.96^b^
**5 d Post-infection**			
*Salmonella* only	168.33 ± 22.73^a^	198.33 ±10.80^a^	311.84 ± 15.89^a^
PZ01 + *Salmonella*	ND	ND	10.13 ± 5.63^e^^f^
JM32 + *Salmonella*	ND	ND	3.87 ± 0.57^f^
JH231 + *Salmonella*	ND	ND	17.50 ± 5.00^e^
JS11 + *Salmonella*	144.67 ± 7.12^a^	66.67 ± 4.08^c^	98.31 ± 10.18^b^
JK22 + *Salmonella*	153.33 ± 10.80^a^	141.67 ± 10.80^b^	52.76 ± 5.23^d^
JM2A1 + *Salmonella*	146.67 ± 11.43^a^	180.00 ± 14.14^a^	71.25 ± 7.50^c^

^a-f^ Different superscripts indicate significant differences among different treatments in the same type of sample on each sampling day (n = 6) (*P*<0.05).

ND: not detectable.

*Salmonella* was detected in livers and spleens from d1 to d5 post-infection for the positive control group. Three strains with higher *in vitro* immunomodulatory properties prevented invasion of *Salmonella* into livers and spleens, with no viable *Salmonella* detected on d1, d3 and d5. Three strains with lower *in vitro* immunomodulatory properties reduced invasion of *Salmonella* in livers and spleens, especially for d1 with no viable *Salmonella* detected except for strain JK22 in the spleen. On d3, JS11 significantly reduced invasion of *Salmonella* in livers and spleens, compared to the positive control. Other 2 strains also significantly reduced invasion of *Salmonella* in livers but they had no effects in spleens. For d5, only JS11 and JM2A1 significantly reduced invasion of *Salmonella* in livers (*P*<0.05), while all three strains with lower *in vitro* immunomodulatory properties had not significantly reduced invasion of *Salmonella* in spleens (*P*>0.05).

### Assay of the cytokines LITAF, IL-1β, IL-6, IL-10 and IL-12 in chick serum

**Fig 3:**
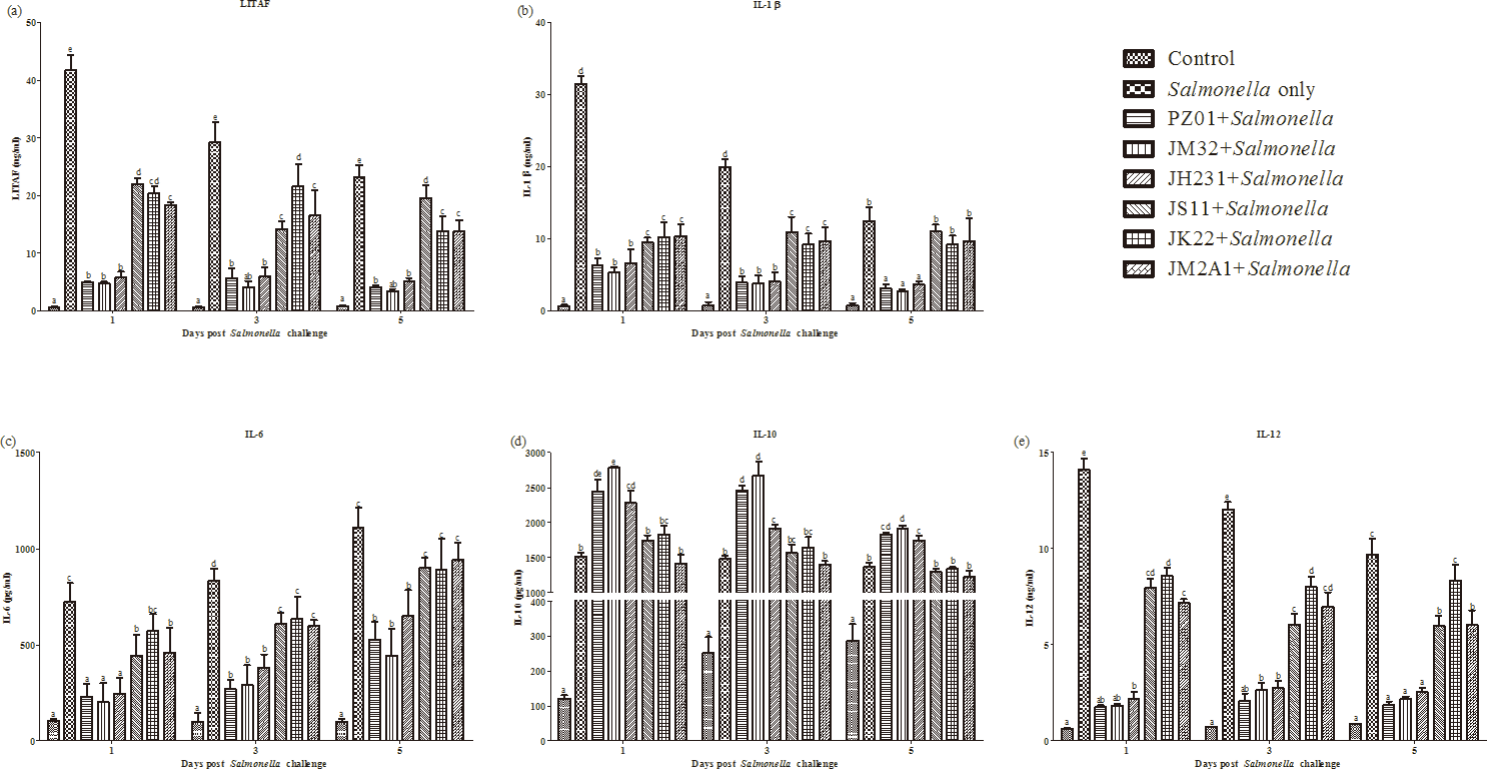
Cytokine levels in sera of chicks gavaged with LAB strains and followed by *Salmonella* challenge. The cytokines (A) LITAF, (B) IL-1β, (C) IL-6, (D) IL-10 and (E) IL-12 were measured by the ELISA methods with specific antibodies of LITAF, IL-1β, IL-6, IL-10 and IL-12 in chicks prepared in our laboratory. Each vertical bar represents the mean ± SD (n = 6). Different letters above bars indicate significant differences among treatments within each sampling day (*P*<0.05).

Blood samples were assayed for pro-inflammatory cytokines LITAF, IL-1β, IL-6, IL-12 and anti-inflammatory cytokine IL-10 (Fig 3). For the positive control group, the highest expression levels of LITAF, IL-1β and IL-12 were observed at 1 day post-infection and gradually decreased afterwards. The opposite was true for IL-6 with increasing concentrations after the infection. However, there was no change of IL-10 after infection.

In comparison with the positive control, three strains with higher *in vitro* immunomodulatory properties (PZ01, JM32 and JH231) significantly reduced LITAF, IL-1β, IL-6 and IL-12 and increased IL-10 levels at all 3 time points. Three strains with lower *in vitro* immunomodulatory activities (JS11, JK22 and JM2A1) also significantly reduced levels of LITAF, IL-1β, IL-6 and IL-12 mainly on d1 and d3 but did not affect IL-10 levels in comparison with the positive control. The abilities of PZ01, JM32 and JH231 to reduce LITAF, IL-1β, IL-6 and IL-12 and to increase IL-10 were significantly greater than those JS11, JK22 and JM2A1.

## Discussion

This study was the first to evaluate effects of LAB on LITAF and IL-12 expression by the spleen mononuclear cells of chickens at the protein level. The reason we chose spleen mononuclear cells was because lactic acid bacteria induced significantly more cytokines in spleen cells than in cecal tonsil cells of chickens [11]. LITAF and IL-12 levels detected in our study were generally higher than those from previous studies using mouse macrophages RAW 264.7 cells [2,3]. TNF-α production was significantly increased by lactic acid bacteria (LAB) strains after their co-culturing with RAW 264.7 cells [2]. A further study from the same research group indicated that viable and heat-killed LAB strains, either individually or in mixture, were able to induce the release of TNF-α and IL-12 from RAW 264.7 cells [3]. Considering the species specialization and higher sensitivities of spleen mononuclear cells from chickens than RAW 264.7 cells, it seems reasonable to use spleen mononuclear cells from chickens to evaluate *in vitro* immunoregulatory activities of lactic acid bacteria for poultry production.

In this study, we demonstrated that cytokines, LITAF, IL-1β, IL-6, IL-10 and IL-12, were involved in the immunity of *Salmonella*-infected chicks. Three strains with higher *in vitro* immunomodulatory properties (PZ01, JM32 and JH231) reduced LITAF, IL-1β, IL-6 and IL-12 and increased IL-10 more efficiently than three other strains with lower *in vitro* immunomodulatory activities (JS11, JK22 and JM2A1). TNF-α is a member of a group of cytokines that stimulate the acute phase reaction in mammal. Although TNF-α has not been found nor described in the chicken genome, LITAF, which is the regulator for TNF-α expression in mammal [18], has been shown to play an important role in the intestinal inflammatory response in chicken [19]. IL-12 is produced by inflammatory myeloid cells and influences the development of T_H_1 cell responses [20]. Similarly, IL-1β is also a major mediator of inflammation and is produced by monocytes, tissue macrophages, enterocytes and other cells [21]. These three cytokines (LITAF, IL-1β and IL-12) indicate an early inflammatory response [22]. The concentrations of LITAF, IL-1β and IL-12 at the protein level were the highest at d1 and gradually decreased afterwards in the positive control group. Similar changes have been reported at the mRNA level by Chen et al. [4]. IL-6 is a multifunctional cytokine. IL-6 gradually increased after the *Salmonella* challenge, similar to the previous findings at the mRNA level for chickens [4,23] and at the protein level for mice [3]. In comparison with the positive control, three strains with higher *in vitro* immunomodulatory activities (PZ01, JM32 and JH231) significantly increased IL-10 levels, while three strains with lower *in vitro* immunomodulatory activities (JS11, JK22 and JM2A1) did not affect IL-10 levels. In addition, this study showed that three strains with higher *in vitro* immunomodulatory properties (PZ01, JM32 and JH231) reduced the levels of *Salmonella* Enteritidis recovered from chick livers, spleens and cecal contents more efficiently than three strains with lower *in vitro* immunomodulatory activities (JS11, JK22 and JM2A1).

The selected six LAB strains for the *in vivo* study showed consistent tolerance to acid and bile salts *in vitro*, suggesting that these six LAB strains could survive the gastrointestinal tract and function effectively [24,25]. Moreover, these six LAB strains were able to inhibit the growth of the pathogenic bacteria. Antimicrobial activities of all six strains might be associated with acidic metabolites such as acetic acid, lactic acid [26] and organic acid [27], or bacteriocins [28,29] and proteinaceous substances [30]. Finally, these six LAB strains were biologically safe due to negative haemolytic activities (data not shown).

In conclusion, compared with strains *E*. *faecium* JS11, *L*. *salivarius* JK22 and *L*. *salivarius* JM2A1 with lower *in vitro* immunomodulatory properties, strains *L*. *plantarum* PZ01, *L*. *salivarius* JM32 and *P*. *acidilactici* JH231 with higher *in vitro* immunomodulatory activities were more effective to reduce *Salmonella* counts in cecal content and decease invasion of *Salmonella* into livers and spleens. These results suggest that *in vitro* immunomodulatory activities could be used as additional parameters to select more effective probiotics for poultry.
